# Short-term safety and immunogenicity of inactivated and peptide-based SARS-CoV-2 vaccines in patients with endocrine-related cancer

**DOI:** 10.3389/fimmu.2022.1028246

**Published:** 2022-10-24

**Authors:** Rui Song, Li Liu, Qingbo Pan, Jin Liu, Jiahe Tan, Juan Deng, Qin Deng, Zijin Lin, Min Chen, Mingli Peng, Hong Ren, Jia Ming

**Affiliations:** ^1^ Department of Infectious Diseases, The Second Affiliated Hospital, Chongqing Medical University, Chongqing, China; ^2^ Department of Breast and Thyroid Surgery, The Second Affiliated Hospital, Chongqing Medical University, Chongqing, China; ^3^ Department of Breast and Thyroid Surgery, The First People’s Hospital of Yibin, Sichuan, China; ^4^ Department of Neurosurgery, The First Affiliated Hospital of Chongqing Medical University, Chongqing, China

**Keywords:** cancer, COVID-19, SARS-COV-2, vaccine, memory B cells

## Abstract

**Background:**

The aim of this study was to explore the short-term safety and immunogenicity of inactivated and peptide-based SARS-CoV-2 vaccines in patients with endocrine-related cancer (ER).

**Methods:**

Eighty-eight patients with ER cancer and 82 healthy controls who had completed a full course of inactivated or peptide-based SARS-CoV-2 vaccines were recruited. Adverse events (AEs) were recorded. Responses to receptor-binding domain IgG antibody (anti-RBD-IgG), neutralizing antibodies (NAbs) and RBD+ memory B cells (MBCs) were evaluated.

**Results:**

Approximately 26.14% (23/88) of patients with ER cancer reported AEs within 7 days, which was comparable to that reported by healthy controls (24.39%, 20/82). Both the overall seroprevalence of anti-RBD-IgG and NAbs was obviously lower in the cancer group (70.45% vs. 86.59%, *P <* 0.05; 69.32% vs. 82.93%, *P <* 0.05, respectively). Anti-RBD-IgG and NAbs titers exhibited similar results, and dropped gradually over time. Patients with ongoing treatment had an attenuated immune response, especially in patients receiving active chemotherapy. The frequency of overall RBD+ MBCs was similar between the two groups, but the percentage of active MBCs was remarkably reduced in patients with ER cancer. Unlike antibody titers, MBCs responses were relatively constant over time.

**Conclusion:**

Inactivated and peptide-based COVID-19 vaccines were well tolerated, but with lower immunogenicity for ER cancer patients. More intensive antibody monitoring and timely booster immunization is recommended for patients with ER cancer presenting disordered subpopulations of RBD+ MBCs.

## Introduction

The severe acute respiratory syndrome coronavirus 2 (SARS−CoV−2) causing the coronavirus disease 2019 (COVID−19) pandemic has threatened individuals worldwide (1). Since the global COVID-19 pandemic, more than 596.87 million individuals have been infected throughout the world, while 6.45 million COVID-19-related deaths have been recorded. (as of 30 August 2022).

The COVID-19 pandemic has had immeasurable consequences on oncological treatment, as well as on the lives of cancer patients, in multiple aspects (2). Cancer patients are at risk for more severe COVID-19 infections and increased mortality due to treatment modalities and medications that can alter immune responses (3). Taghizadeh-Hesary et al. discovered that COVID-19 patients with a history of cancer had a higher rate of mechanical ventilation and mortality than patients without a history cancer (4). Furthermore, according to Javadinia et al., more than 20% of cancer patients may have asymptomatic COVID-19 (5). Several drugs are being considered for COVID-19 therapy, including antiviral drugs (such as molnupiravir, paxlovid, and remdesivir), anti-inflammatory drugs (such as colchicine and methylprednisolone), and adjunct drugs (such as antibiotics, anticoagulants, and vitamins) (6). However, there is currently no specific treatment available that can cure COVID-19. Therefore, vaccination is critical for the prevention of COVID-19, especially for cancer patients (7). More recently, several reports suggested that the reactivity of the SARS-COV-2 vaccines is compromised in patients with cancer, HBV or HIV infection, older patients, and in those receiving chemotherapy (8–11). Meanwhile, healthy controls are included in few of these studies and are limited in numbers. Importantly, most studies focus mainly on antibody responses, with little mention of durable humoral immunity responses, which are typically mediated by memory B cells (12). There are numerous types of vaccination protocols being evaluated and developed. Inactivated COVID-19 vaccines (BBIBP-CorV, CoronaVac) and RBD-based protein subunit vaccines (ZF2001) have been adopted more frequently in China (13). However, data on the safety and humoral immune responses of inactivated and peptide-based SARS-CoV-2 vaccines in patients with endocrine-related (ER) cancer are still lacking.

Therefore, we designed a prospective observational study to evaluate receptor-binding domain IgG antibody, neutralizing antibodies, and RBD+ memory B cells, and to monitor adverse events (AEs) in patients with ER cancer and healthy controls 20-115 days after the full course of SARS-CoV-2 vaccines.

## Materials and methods

### Participants and study design

In this prospective observational research, adult patients (age >18 years) diagnosed with ER cancer (thyroid or breast cancer) and healthy adult controls, who received full doses of COVID-19 vaccination (CoronaVac/BBIBP-CorV) or ZF2001, were consecutively recruited from the Second Affiliated Hospital of Chongqing Medical University. Participants with the following conditions were not included: history of autoimmune diseases, pregnancy, or COVID-19 infection. From July 2021 to April 2022, 170 participants were enrolled in this study. Among them, 88 were ER cancer patients and 82 were healthy controls.

AEs occurred within 7 and 30 days were recorded by questionnaires. All AEs were documented and classified using the China National Medical Products Administration scale (version 2019). The investigators evaluated AEs related to vaccination. serious AEs were monitored for up to 1 year. A questionnaire or an electronic medical record was used to collect demographic characteristics, cancer therapy history, medical records, and clinical data.

In this cross-sectional study, serum anti-RBD-IgG, NAb, and RBD+ MBCs frequencies were evaluated for all participants 20–115 days after the last immunization dose. During follow-up, the dynamic variations of serum antibody titers and the frequencies of RBD+ MBCs were also evaluated. We also monitored antibody titers of participants who had received a booster vaccination (**Supplementary Figure 1**).

This ongoing study was approved by the Ethics Committee of the Second Affiliated Hospital of Chongqing Medical University and followed the ethical guidelines of the Declaration of Helsinki. Written informed consent was obtained from all participants.

### SARS-CoV-2 antibody test

Based on the manufacturer’s directions, plasma samples were taken for detecting antibody titers using capture chemiluminescence immunoassays by MAGLUMITM X8 (China, Snibe). NAbs tests had 100% sensitivity and 100% specificity, while the anti-RBD-IgG tests had 100% sensitivity and 99.6% specificity for the diagnosis of COVID-19. The cut-off value were 1 AU/mL and 0.15 µg/mL for anti-RBD-IgG and NAbs, respectively.

### RBD+ memory B cell responses

Streptavidin BV421 (405225; Biolegend, California, CA, USA) and biotinylated SARS-CoV-2 Spike RBD protein ((40592-V08H2-B; Sino Biological, Beijing, China) were incubated for 1 hour at 4°C to create the antigen probe. Peripheral blood mononuclear cells (PBMCs) were separated from heparinized blood base on the manufacturer’s recommendations using Histopaque density gradient centrifugation (10771, Sigma-Aldrich). After rinsing with FACS buffer (phosphate-buffered saline with 2% fetal bovine serum), staining was performed for 30 min at 4°C with an antigen probe (1:33.3) and the following binding antibodies: anti-human CD3 (300430, Biolegend), anti-human CD19 (302212, Biolegend), anti-human CD21 (354918, Biolegend), and anti-human CD27 (356406, Biolegend). These antibodies were added in 1:50 volume. After staining, cells were rinsed and resuspended in 150 µL FACS buffer. Subsequently, samples were evaluated using a flow cytometer (CytoFLEX, Beckman Coulter). FlowJo software version 10.0.7r2 was used for data analysis.

### Statistical analysis

Appropriate methods were used for statistical analysis based on the type of data. Continuous variables were compared by Mann–Whitney U test for two groups. Categorical variables were compared by Chi-square or Fisher’s exact test. The Spearman’s rank correlation was used to evaluate the correlation between two antibodies. Clinical parameters related to antibody titers were found using simple and multivariate regression analysis. Categorical variables were reported as numbers (%), whereas continuous variables were presented as the median (IQR). *P <* 0.05 was considered statistically different. SPSS (IBM, 24.0.0) was used to analyze statistics. GraphPad Prism software (9.2.0) was used to make graphs.

## Results

### Characteristics of participants

From July 2021 to April 2022, 170 participants were recruited for this observational study. Of these, 88 were patients with ER cancer and 82 were healthy controls without tumors. As shown in **Table 1**, the median age was 44 years (interquartile range [IQR]: 34.25–55.75 years) in patients with ER cancer and 46 years (IQR: 40.00–52.00 years) in healthy controls. Most of the participants were women: (84.09% [74/88] among cancer patients and 82.93% [68/82] among healthy controls). The median time after vaccination was 68 days (IQR: 45–86 days) and 64 days (IQR: 45–69 days) for patients with ER cancer and healthy controls, respectively. There were no significant differences in the body mass index (BMI) or vaccine types between the two groups.

**Table 1:** The demographic characteristics of participants.

Variables	ER cancer patients (n = 88)	Healthy controls (n = 82)	P-value
Age, (years)	44 (34.25-55.75)	46 (40.00-52.00)	0.159
Gender, female, n (%)	74 (84.09)	68 (82.93)	1.000
Days after 2^nd^ or 3^rd^ dose vaccination, (days)	68 (45-86)	64 (45-69)	0.395
BMI	23.74 (21.26-25.91)	22.88 (21.19-26.02)	0.652
Vaccine type			
BBIBP-CorV, n (%)	30 (34.09)	24 (29.27)	0.877
CoronaVac, n (%)	25 (28.41)	23 (28.05)	
Mixed vaccination, n (%)	21 (23.86)	21 (25.61)	
ZF 2001, n (%)	12 (13.64)	14 (17.07)	
Cancer type			
Breast cancer, n (%)	40 (45.45)	/	/
Thyroid cancer, n (%)	48 (54.55)	/	/
Anti cancer therapy			
Active treatment^#^, n (%)	59 (67.05)	/	
Previous treatment, n (%)	15 (17.04)	/	/
Treatment naïve, n (%)	14 (15.91)	/	
Laboratory result			
RBC (10^12^/L)	4.40 (4.15-4.70)	4.61 (4.40-4.86)	0.034
HB (g/L)	132.00 (127.00-142.00)	136.00 (128.00-142.00)	0.329
WBC (10^9^/L)	5.91 (5.01-6.93)	5.36 (4.61-6.43)	0.093
PLT (10^9^/L)	247 (197-288)	217 (177-260)	0.016
LYC (10^9^/L)	1.72 (1.48-2.09)	1.74 (1.46-2.18)	0.855
ALB (g/L)	44.60 (41.25-46.53)	45.80 (44.40-47.20)	0.031
ALT (U/L)	18 (13-27)	23 (17-29)	0.096
AST (U/L)	21 (18-26)	22 (17-26)	0.821
TSH (uIU/ml, thyroid cancer)	1.33 (0.53-2.15)	/	/
Thyroglobulin (IU/ml, thyroid cancer)	23.00 (19.10-32.85)	/	/

^#^Patients undergoing anti-cancer therapy within 6 months before the first dose vaccination. BMI, body mass index; RBC, red blood cell; HB, hemoglobin; WBC, white blood cell; PLT, platelet; LYC, lymphocyte; ALB, albumin; ALT, alanine aminotransferase; AST, aspartate aminotransferase; TSH, thyroid stimulating hormone.

Of the 88 patients with ER tumors, 48 had thyroid cancers and 40 had breast cancers. Among the 88 patients with ER cancer, 59 (67.05%) had received active anticancer treatment within 6 months before the first dose of vaccination, 15 (17.04%) had a history of anticancer therapy, and 14 (15.91%) had never undergone anticancer therapy. Of the 59 patients with ongoing treatment, 27 had thyroid cancer and 22 breast cancer. Additionally, the results of clinical laboratory tests, such as red blood cell (RBC), platelet (PLT) and albumin (ALB), were different between two groups (**Table 1**).

### Safety of SARS-COV-2 vaccines in patients with ER cancer

In general, AEs within 7 days were reported in 26.14% (23/88) of cancer patients, which was comparable to healthy controls (24.39%, 20/82) (**Table 2**). In detail, the common local AEs (>3%) in cancer patients were pain (23.86%), swelling (15.91%), redness (12.50%), itch (13.64%), and induration (12.50%) at the injection site, the usual systemic AEs were muscle pain (23.86%), dizziness (13.64%), and headache (9.09%). The most common AEs in healthy controls was muscle pain (21.95%). More AEs occurred when the observation period was prolonged to 30 days (**Supplementary Table 1**). Briefly, 28.41% (25/88) of cancer patients and 26.83% (22/82) of healthy controls reported AEs within 30 days. Most AEs within 30 days were mild, no serious AEs were observed. At the end of this study, five patients with ER cancer had been vaccinated for 1 year and no more or more serious vaccine-related adverse effects were observed. Taken together, SARS-COV-2 vaccines in patients with ER tumors were well tolerated.

**Table 2:** Adverse events of COVID-19 vaccination in enrolled participants.

Adverse events within 7 days	ER cancer patients (n = 88)	Healthy controls (n = 82)	P-value
Overall adverse events, n (%)	23 (26.14)	20 (24.39)	0.861
Local adverse events			
Pain, n (%)	21 (23.86)	17 (20.73)	0.713
Swelling, n (%)	14 (15.91)	11 (13.41)	0.671
Redness, n (%)	11 (12.50)	10 (12.20)	1.000
Itch, n (%)	12 (13.64)	7 (8.54)	0.337
Induration, n (%)	11 (12.50)	6 (7.32)	0.312
Systemic adverse events			
Muscle pain, n (%)	21 (23.86)	18 (21.95)	0.856
Pruritus, n (%)	2 (2.27)	1 (1.22)	1.000
Rash, n (%)	1 (1.14)	1 (1.22)	1.000
Fatigue, n (%)	1 (1.14)	0 (0)	1.000
Drowsiness, n (%)	1 (1.14)	0 (0)	1.000
Dizziness, n (%)	12 (13.64)	8 (9.76)	0.482
Headache, n (%)	8 (9.09)	7 (8.54)	1.000
Rhinorrhea, n (%)	2 (2.27)	1 (1.22)	1.000
Laryngeal pain, n (%)	1 (1.14)	0 (0)	1.000
Fever, n (%)	1 (1.14)	0 (0)	1.000
Chill, n (%)	1 (1.14)	0 (0)	1.000
Cough, n (%)	0 (0)	0 (0)	1.000
Inappetence, n (%)	0 (0)	0 (0)	1.000
Abdominal pain, n (%)	0 (0)	0 (0)	1.000
Abdominal distension, n (%)	1 (1.14)	0 (0)	1.000
Diarrhea, n (%)	1 (1.14)	0 (0)	1.000
Hepatalgia, n (%)	0 (0)	0 (0)	1.000
Nausea, n (%)	0 (0)	1 (1.22)	0.482
Chest distress, n (%)	1 (1.14)	0 (0)	1.000
Constipation, n (%)	0 (0)	0 (0)	1.000

### Antibody responses to the SARS-CoV-2 vaccines in patients with ER cancer

**Figure 1:**
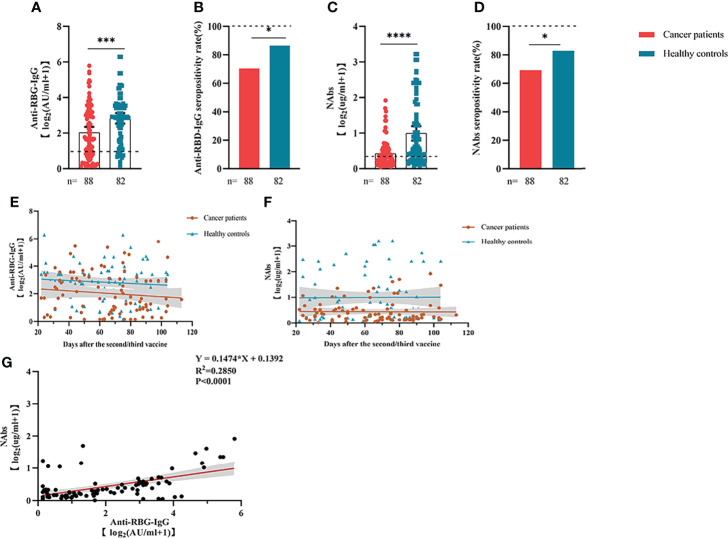
Responses of antibodies and RBD+ B cells to the SARS-COV-2 vaccines. The responses of antibodies **(A–D)** in healthy controls and patients with ER cancer. Change in antibody titers over time **(E, F)**. The correlation between anti-RBD-IgG and NAbs **(G)**. ER, endocrine-related, RBD, receptor-binding domain, NAbs, neutralizing antibodies, MBCs, memory B cells. *p < 0.05, ***p < 0.001, ****p < 0.0001.

Anti-RBD-IgG seroprevalence in patients with ER cancer was 70.45% overall, which was significantly lower than the overall seroprevalence in healthy groups (86.59%, *P <* 0.05) (**Figure 1B**). Anti-RBD-IgG levels were also lower in patients with ER-tumors. (6.43 IU/mL [IQR: 0.76-7.64] vs. 10.05 IU/mL [2.56–10.64], *P <* 0.001) (**Figure 1A**). Given the importance of NAbs in defense against live SARS-CoV-2, NAbs titers were also investigated in the present study. Similar to anti-RBD-IgG, the positive rate of NAbs was less detected in patients with ER cancer (69.32% vs. 82.93%, *P <* 0.05) (**Figure 1D**). NAb titers were also significantly lower in patients with ER cancer (0.42 µg/mL [0.11–0.44] vs. 1.53 µg/mL [0.21–1.82], *P <* 0.0001) (**Figure 1C**). A linear model based on data 20–115 days after full-course vaccination was used to better assess the durability of circulating antibody titers. The antibody titers in both groups, especially anti-RBD-IgG, exhibited a continual reduction, even though the titers of anti-RBD-IgG and NAbs titers in healthy controls were higher than in cancer patients. (**Figures 1E, F**). As expected, the two antibodies were well correlated (r^2 =^ 0.2850, *P <* 0.001) (**Figure 1G**).

**Figure 2:**
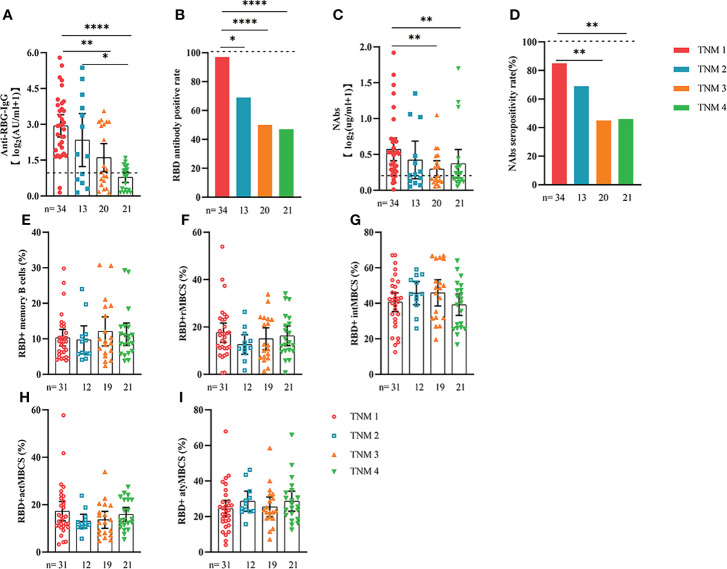
Responses of antibodies and RBD+ B cells to the SARS-COV-2 vaccines. The responses of antibodies **(A–D)** and RBD+ MBCs **(E–I)** in patients with ER cancer of different TNM grade. ER, endocrine-related, RBD, receptor-binding domain, NAbs, neutralizing antibodies, MBCs, memory B cells. *p < 0.05, **p < 0.01, ****p < 0.0001.

**Figure 3:**
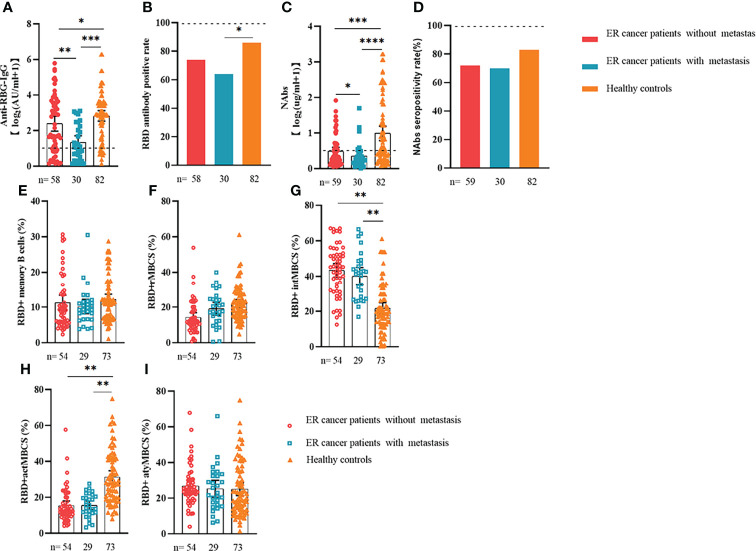
Responses of antibodies and RBD+ B cells to the SARS-COV-2 vaccines. Responses of antibodies **(A–D)** and RBD+ MBCs **(E–I)** in patients with ER cancer with/without metastasis. ER, endocrine-related, RBD, receptor-binding domain, NAbs, neutralizing antibodies, MBCs, memory B cells. *p < 0.05, **p < 0.01,***p < 0.001, ****p < 0.0001.

**Figure 4:**
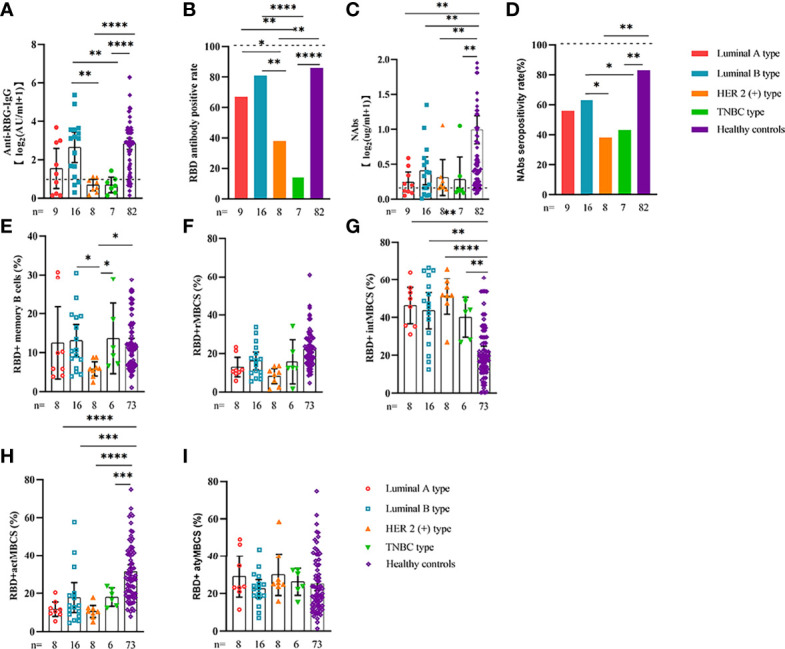
Responses of antibodies and RBD+ B cells to the SARS-COV-2 vaccines. Responses of antibodies **(A–D)** and RBD+ MBCs **(E–I)** in breast cancer patients of different genotype. ER, endocrine-related, RBD, receptor-binding domain, NAbs, neutralizing antibodies, MBCs, memory B cells. *p < 0.05, **p < 0.01,***p < 0.001, ****p < 0.0001.

**Figure 5:**
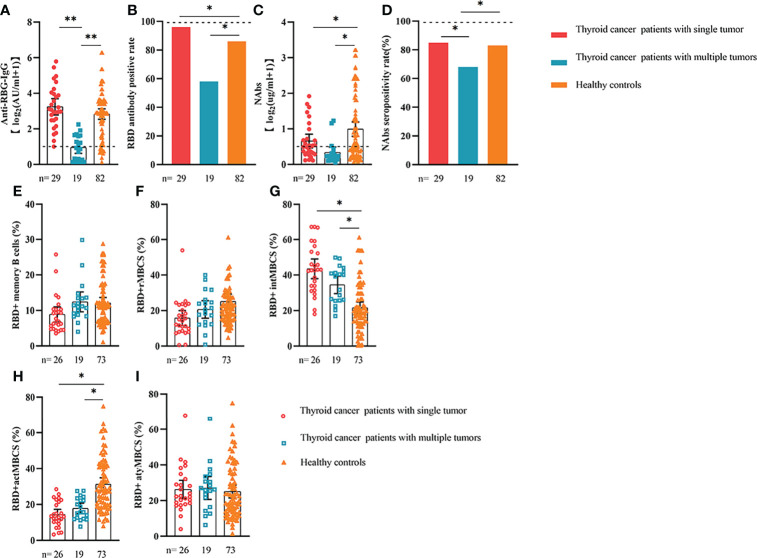
Responses of antibodies and RBD+ B cells to the SARS-COV-2 vaccines. The responses of antibodies **(A–D)** and RBD+ MBCs **(E–I)** in patients with thyroid cancer with single/multiple tumors. ER, endocrine-related, RBD, receptor-binding domain, NAbs, neutralizing antibodies, MBCs, memory B cells. *p < 0.05, **p < 0.01.

**Figure 6:**
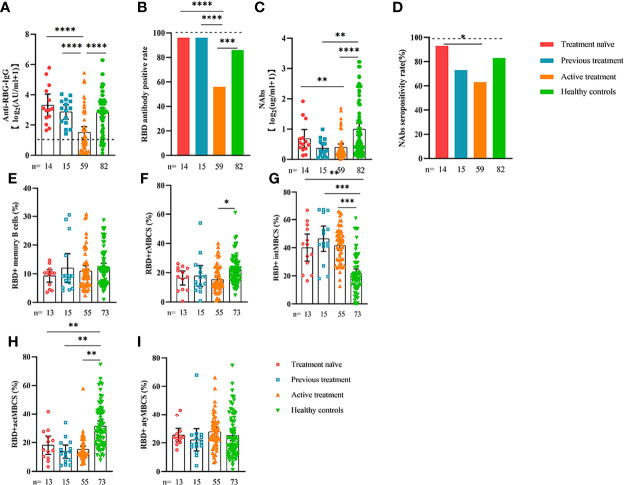
Responses of antibodies and RBD+ B cells to the SARS-COV-2 vaccines. Responses of antibodies **(A–D)** and RBD+ MBCs **(E–I)** in patients with ER cancer of different treatment types. ER, endocrine-related, RBD, receptor-binding domain, NAbs, neutralizing antibodies, MBCs, memory B cells. *p < 0.05, **p < 0.01,***p < 0.001, ****p < 0.0001.

**Figure 7:**
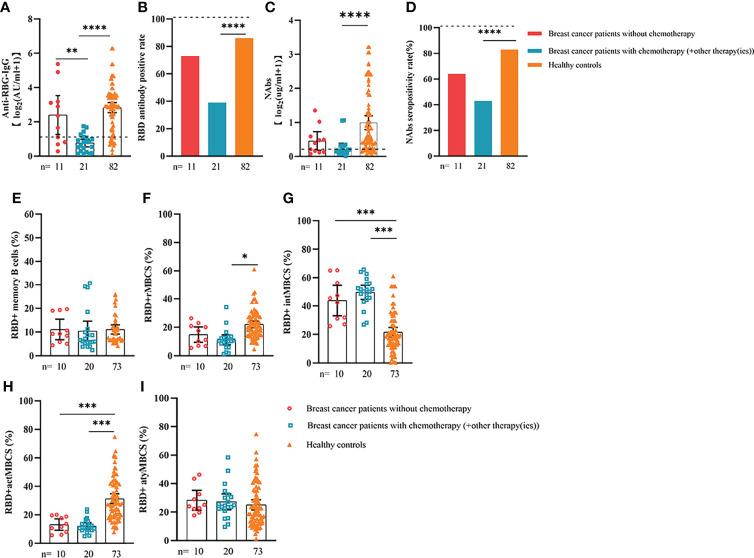
Responses of antibodies and RBD+ B cells to the SARS-COV-2 vaccines. The responses of antibodies **(A–D)** and RBD+ MBCs **(E–I)** in patients with breast cancer treated with/without chemotherapy. ER, endocrine-related, RBD, receptor-binding domain, NAbs, neutralizing antibodies, MBCs, memory B cells. *p < 0.05, **p < 0.01,***p < 0.001, ****p < 0.0001.

Comparable results of antibody responses were observed in the age and sex subgroup analysis (**Supplementary Figures 3**, **4**). Young (age <50 years) and female patients with ER cancer appeared to have better anti-RBD-IgG and NAb responses, but the difference was not significant. In subgroup analysis, we found that patients with ER cancer, with metastasis, higher ASA score or TNM grade, confirmed diagnosis of breast cancer, multiple thyroid tumors, HER2+ or triple-negative breast cancer (TNBC) genotype, and recipients of active anticancer treatment (particularly chemotherapy) had extremely lower anti-RBD-IgG titers (**Figures 2**–**7**; **Supplementary Figures 2**, **5**). The responses of NAbs responses showed a trend similar to that of anti-RBD-IgG. Next, we sought to assess factors associated with inferior antibody responses in patients with ER cancer. As indicated in **Table 3**, the factors obviously related to the lower anti-RBD-IgG titers were days after full-course vaccination, TNM, ASA score, and active chemotherapy. Patients with a longer postvaccination interval, higher ASA or TNM grade, and those who had received chemotherapy showed lower antibody titers. In addition, factors associated with the poor responses to NAbs in patients with ER cancer showed the same results (**Supplementary Table 2**). The clinical characteristics of patients with ER cancer who were negative for anti-RBD-IgG or NAb are presented in **Supplementary Table 3**.

**Table 3:** Simple and multiple regression analysis to identify risk factors of low anti-RBD-IgG titers in endocrine-related cancer patients.

Variables	Simple linear regression		Multiple linear regression	
	β value (95% CI)	P-value	β value (95% CI)	P-value
Age (years)	-0.016 (-0.017, 0.018)	0.078		
Gender (female)	-0.677 (-0.990, 1.105)	0.315		
**Days after full-course vaccination**	**-0.021 (-0.043, -0.009)**	**0.000**	**-0.011 (-0.024, -0.005)**	**0.000**
Comorbidity(ies) (no)	-0.021 (-0.024, 0.001)	0.105		
**TNM (TNM 4)**	**1.236 (1.163, 2.211)**	**0.028**	**1.236 (1.163, 2.211)**	**0.028**
**ASA (ASA 3)**	**1.226 (1.033, 2.249)**	**0.035**	**1.207 (1.503, 2.279)**	**0.033**
Active treatment (no)	0.219 (0.163, 2.249)	0.052		
**Active chemotherapy (no)**	**-0.826 (-1.222, -0.109)**	**0.000**	**-0.800 (-1.091, -0.111)**	**0.000**

TNM, Tumor Node Metastasis; ASA, American Society of Anesthesiologists. Bold values: statistically significant.

### RBD+ B cell responses in patients with ER cancer

We assessed the responses of RBD+ MBCs in patients with ER cancer. The gating strategy was shown in **Supplementary Figure 9**. Surprisingly, there were not statistically significant differences in the frequency (percentage of total B cells) of RBD+ MBCs between patients with ER cancer and healthy controls (10.88% vs. 12.11%, *P* > 0.05) (**Supplementary Figure 7**). To better understand the functional phenotypes of RBD+ MBCs, four subsets of responses from RBD+ MBCs were analyzed, including resting MBCs (rMBCs, CD21+CD27+), activated MBCs (actMBCs, CD21-CD27+), intermediate MBCs (intMBCs,CD21-CD27-), and atypical MBCs (atyMBCs,CD21+CD27-). Interestingly, the percentage of rMBCs and actMBCs decreased in the cancer group (rMBCs: 15.97% vs. 22.02%, *P <* 0.05; actMBCs: 15.48% vs. 31.33%, *P <* 0.01). In contrast, the percentage of intMBCs and atyMBCs was higher in the cancer group (intMBCs: 42.22% vs.21.57%, *P <* 0.001; atyMBCs: 26.33% vs. 25.08%, p>0.05). (**Supplementary Figure 7**).

**Figure 8:**
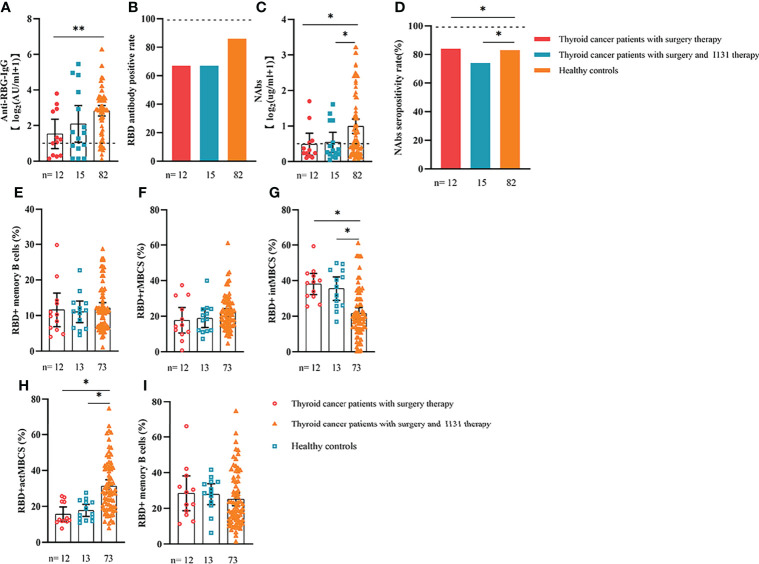
Responses of antibodies and RBD+ B cells to the SARS-COV-2 vaccines. Responses to antibodies **(A–D)** and RBD+ MBCs **(E–I)** in patients with thyroid cancer treated with/without I131 therapy. ER, endocrine-related, RBD, receptor-binding domain, NAbs, neutralizing antibodies, MBCs, memory B cells. *p < 0.05, **p < 0.01.

We also analyzed the durability of circulating RBD+ MBCs from 20 to 115 days after full-course vaccination. As shown in **Supplementary Figure 7**, the frequency of actMBCs was similar to that at the initial time point, and then increased over time in healthy controls, which was slightly decreased in patients with ER cancer. Furthermore, the percentage of RBD+ MBCs and the other three subsets of MBCs in both groups were relatively stable at the interval time. In subgroup analysis, we found that patients with ER cancer diagnosed as breast cancer, with metastasis, thyroid cancer patients with multiple tumors or I131 therapy, breast cancer patients diagnosed as HER2+ or TNBC genotype or treated with chemotherapy showed a remarkably lower actMBCs and higher intMBCs or atyMBCs (**Figures 3**-**5**, **7**, **8**; **Supplementary Figure 2**).

### Follow-up and booster vaccine in ER cancer patients

**Figure 9:**
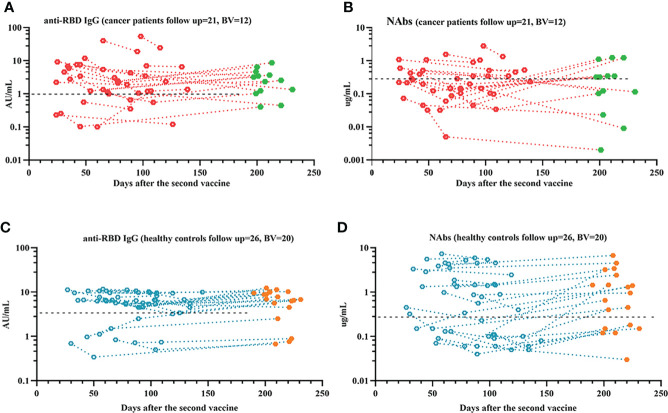
Responses of antibodies to the SARS-COV-2 vaccines over time. The responses of antibodies **(A–D)** over time. The red or blue dots represent samples recorded prior to booster vaccine, and the green or orange dots represent samples recorded after booster vaccine. ER, endocrine related, RBD, receptor-binding domain, NAbs, neutralizing antibodies.

In this study, 47 people completed the follow-up, which is still ongoing. Our results showed that anti-RBD-IgG and NAb titers significantly decreased during follow-up time in both patients with ER cancer and healthy controls. (**Figure 9**). However, when a booster shot was given, most individuals presented a significant increase in antibody titers (**Figure 9**). Additionally, we investigated changes in RBD+ MBCs and found that actMBCs markedly declined over time in most participants, although the frequencies of total RBD+ MBCs and other subpopulations remained rather stable. (**Supplementary Figure 8**)

## Discussion

We investigated the safety and humoral immune responses of the SARS-CoV-2 vaccine in healthy controls and patients with ER cancer in this prospective observational study. Our results showed that the SARS-CoV-2 vaccines were well tolerated in patients with ER cancer. No serious vaccine-related AEs occurred. Anti-RBD-IgG and NAb responses were lower in patients with ER cancer, especially in breast patients receiving active chemotherapy treatment. Comparable results of actMBCs responses were observed. In both groups, antibody responses were reduced over time; however, RBD+ MBCs responses were relatively stable. Lastly, a longer time after full-course vaccination, higher grade of TMN or ASA, and ongoing chemotherapy were related to poor antibody responses in patients with ER cancer.

Data regarding the safety and humoral immune responses of SARS-CoV-2 vaccines in ER cancer participants were limited. Therefore, we first evaluated the short-term safety profile, and the results indicated that the overall incidence of AEs in patients with ER cancer within 7 days was similar to that of healthy controls, which was consistent with earlier studies (14–16). However, it was higher than our previous study in patients with gastrointestinal cancer (26.14% vs. 22.29%) (17), which suggests that different types of vaccines and cancers can contribute to this discrepancy. Furthermore, most of the AEs were mild and no vaccine-related serious AEs were reported in this study, which was consistent with earlier studies (9, 18, 19). Although 59 patients were receiving ongoing anticancer treatments (surgery, I131 therapy, immunotherapy, endocrinotherapy, or chemotherapy), no serious AEs were recorded, further highlighting the good tolerance of the SARS-COV-2 vaccines. However, in our study, only five patients had data on AEs after 1 year of vaccination. According to Tom T. Shimabukuro, a 1-year follow-up is sufficient to assess most acute and delayed-onset AEs of interest for vaccine safety, but it is not sufficient to assess conditions with an onset multiple years following exposure (20). Therefore, we are currently closely monitoring participants for vaccine-related AEs.

Next, we evaluated antibody responses between patients with ER tumors and healthy individuals. Our findings were in agreement with earlier studies (9, 21) and showed that serum anti-RBD-IgG and NAb levels in patients with ER cancer were significantly lower after a complete course of immunization. The overall seropositivity rate of anti-RBD-IgG and NAb in cancer patients was significantly lower than in healthy controls, and it was also lower than in previous studies of mRNA vaccines and inactivated vaccines (21), which could be attributed to the difference in vaccine type, cancer type, antibody testing kits, and interval time after full-course vaccination. Moreover, a continuously decay of antibody titers was observed with time in both ER cancer patients and healthy controls, which was similar to the results of previous longitudinal studies (17, 22, 23). Furthermore, the decrease in anti-RBD-IgG was particularly obvious in the cancer population; the results indicated that vaccine protection in patients with ER cancer may be weakened faster than in healthy controls. Therefore, greater attention should be paid to this special population.

Based on multiple linear regression, we found that a longer time after vaccination, a higher ASA score or TNM grade, and ongoing chemotherapy were adverse factors for worse antibody responses. A higher ASA or TNM grade usually indicated higher risks for anesthesia and poorer clinical conditions (17). Our findings were consistent with a previous study that found that cancer patients with poor ECOG PS (>2) had a higher chance of invalid vaccination (21). Treatment modalities have been proven to influence antibody titers in cancer patients (24, 25). In our study, patients undergoing treatment showed the poorest antibody responses, which was similar to that reported by previous studies (26, 27). Further subgroup analysis revealed that antibody levels in breast cancer patients receiving active chemotherapy were extremely lower than those of all other groups, which corresponded to previous studies (28, 29). We did not examine the impact of molecular-targeted therapy, endocrinotherapy, or immunotherapy on vaccine effectiveness because there were few patients in this study who received these regimens.

We also evaluated the RBD+ MBCs responses in cancer patients. MBCs are the pivot element for quick and valid antibody responses in case of reinfection, actMBCs are likely to become antibody-secreting cells, and their frequency correlates strongly with serum antibody levels (30). In contrast, numerous studies have pointed to the adverse correlation of intMBCs and atyMBCs with antibody titers (31, 32). Although no differences in the frequencies of total MBCs of two groups were observed in our study, patients with ER tumors had significantly lower frequencies of actMBCs and higher frequencies of intMBCs and atyMBCs. Comparable results were found in breast cancer patients, thyroid cancer patients with multiple tumors or in those receiving I^131^ therapy, breast cancer patients diagnosed with the HER2+ or TNBC genotype or those being treated with chemotherapy, and patients with metastasis. This may help partially explain why humoral responses to COVID-19 vaccinations were weakened in cancer patients.

In addition to B cell-mediated humoral immunity, T cell immune responses also play a key role in vaccine-mediated protection. Cytotoxic CD8+ T lymphocytes may limit the spread of infectious agents by recognizing and killing infected cells (6). CD4+ T-helper lymphocytes (Th) support potent B-cell activation and differentiation into antibody-secreting cells and have been identified as directly controlling antibody responses and mediating an adjuvant activity (6). Recent reports have highlighted the discordance of the humoral response with T cell responses after COVID-19 vaccination in cancer patients in contrast to healthy controls, where responses are better coordinated (33). Furthermore, many studies have found that decreased magnitudes of T cell responses in cancer patients compared to healthy controls can be observed by quantification of interferon-γ (IFN-γ) using enzyme-linked immunospot (ELISPOT) assays or using flow-cytometry to detect T cell quantification assays or cytokine-producing T cells (34, 35). Thus, T cell response should be considered when evaluating the level of protection generated through COVID-19 vaccination.

Finally, we monitored long-term changes in vaccine antibodies and humoral immunity. During the follow-up after receiving the full course of immunization, the serum anti-RBD-IgG and NAbs levels significantly declined over time. Our results were consistent with earlier studies (13, 36) and showed that serum antibody titers increased remarkably after the administration of booster vaccination. The frequency of actMBCs decreased with time in most individuals, while the frequencies of the total and other subpopulations of MBCs remained relatively unchanged. A stable frequency of RBD+ MBCs might be a necessary condition for a functional booster vaccine.

There are several limitations in our study that should be considered. First, only 47 and 32 participants completed follow-up and had booster vaccine, respectively. Therefore, more patients with ER cancer should be included in future studies. Second, other immune cells also played a key role in antibody responses to the SARS-COV-2 vaccines, and the functions of these cells should be further investigated. Third, there are numerous types of endocrine tumors, such as pituitary adenoma, prostate cancer, and ovarian cancer. We only recruited patients with thyroid and breast cancer, and more other endocrine tumors need to be studied. Despite these limitations, we believe that our findings are very meaningful to clinicians.

## Conclusion

Our data revealed that inactivated and peptide-based SARS-CoV-2 vaccines are well tolerated, and presented no short-term vaccine-related serious AEs in patients with ER cancer. However, antibody responses and durable humoral immune responses were impaired, particularly in patients with active chemotherapy. Therefore, for this special population, routine testing of serum antibody titers and booster vaccines should be given priority.

## Data availability statement

The original contributions presented in the study are included in the article/**Supplementary Materials**. Further inquiries can be directed to the corresponding authors.

## Ethics statement

The studies involving human participants were reviewed and approved by Ethics Committee of the Second Affiliated Hospital of Chongqing Medical University. The patients/participants provided their written informed consent to participate in this study.

## Author contributions

Conception and design of the study: RS, LL, QP, JM, HR. Participant recruitment: RS, LL, QP, JD, QD, ZL, JL, MP. Experiment execution: RS, QP, MC. Acquisition and analysis of data: RS, LL, QP, JT, JD, QD, ZL. Drafting the article or revising it critically for important intellectual content: RS, LL. Final approval of the version to be submitted: JM, HR. All authors contributed to the article and approved the submitted version.

## Funding

This work is supported by the National Science and Technology Major Project of China (2017ZX10202203-007, 2017ZX10202203-008, 2018ZX10302206-003) and the Postgraduate research and innovation projects of Chongqing Municipal Education Commission (CYS20197).

## Acknowledgments

We thank the health care center and department of clinical laboratory of the Second Affiliated Hospital, Chongqing Medical University for their support.

## Conflict of interest

The authors declare that the research was conducted in the absence of any commercial or financial relationships that could be construed as a potential conflict of interest.

## Publisher’s note

All claims expressed in this article are solely those of the authors and do not necessarily represent those of their affiliated organizations, or those of the publisher, the editors and the reviewers. Any product that may be evaluated in this article, or claim that may be made by its manufacturer, is not guaranteed or endorsed by the publisher.

## Supplementary material

Supplementary Figure 1 Study flow chart.

Supplementary Figure 2 Responses of antibodies and RBD+ B cells to the SARS-COV-2 vaccines. The responses of antibodies **(A–D)** and RBD+ MBCs **(E–I)** in breast or thyroid cancer and healthy controls. RBD, receptor-binding domain, NAbs, neutralizing antibodies, MBCs, memory B cells.

Supplementary Figure 3 Responses of antibodies and RBD+ B cells to the SARS-COV-2 vaccines. Responses of antibodies **(A–D)** and RBD+ MBCs **(E–I)** in patients with ER cancer and healthy controls stratified by sex. ER, endocrine-related, RBD, receptor-binding domain, NAbs, neutralizing antibodies, MBCs, memory B cells.

Supplementary Figure 4 Responses of antibodies and RBD+ B cells to the SARS-COV-2 vaccines. The responses of antibodies **(A–D)** and RBD+ MBCs **(E–I)** in patients with ER cancer stratified by age. ER, endocrine-related, RBD, receptor-binding domain, NAbs, neutralizing antibodies, MBCs, memory B cells.

Supplementary Figure 5 Responses of antibodies and RBD+ B cells to the SARS-COV-2 vaccines. Responses of antibodies **(A–D)** and RBD+ MBCs **(E–I)** in patients with ER cancer stratified by ASA scores. ER, endocrine-related, RBD, receptor-binding domain, NAbs, neutralizing antibodies, MBCs, memory B cells.

Supplementary Figure 6 Responses of antibodies and RBD+ B cells to the SARS-COV-2 vaccines. Responses of antibodies **(A–D)** and RBD+ MBCs **(E–I)** in patients with ER cancer with/without comorbidity(ies). ER, endocrine-related, RBD, receptor-binding domain, NAbs, neutralizing antibodies, MBCs, memory B cells.

Supplementary Figure 7 Responses of RBD+ B cells. The frequencies of RBD+ MBCs in ER cancer patients and healthy controls **(A, C, E, G, I)**. The change of frequencies of RBD+ MBCs **(B, D, F, H, J)** over time in ER cancer patients and healthy controls. ER, endocrine-related, RBD, receptor-binding domain, MBCs, memory B cells.

Supplementary Figure 8 Responses of RBD+ B cells over time. Dynamic changes of RBD+ MBCs and their subpopulations over time in ER patients and healthy controls. The red dots represent patients with ER cancer, and the blue dots represent healthy controls. RBD, receptor-binding domain, MBC, memory B cells, ER, endocrine related.

Supplementary Figure 9 Gating strategy for the target cell population. RBD, receptor-binding domain.
