# Supplementary material for: Short-term safety and immunogenicity of inactivated and peptide-based SARS-CoV-2 vaccines in patients with endocrine-related cancer
Source: Front Immunol. 2022 Oct 24;13:1028246. doi: 10.3389/fimmu.2022.1028246 (PMC9637626; doi:10.3389/fimmu.2022.1028246)
Supplement: Supplementary file 1 [file Table_1.docx]

| Supplementary Table 1. Adverse events of COVID-19 vaccination in enrolled participants. | | | |
| --- | --- | --- | --- |
| Adverse events within 30 days | Cancer patients  (n=88) | Healthy controls  (n=82) | P-value |
| Overall adverse events, n (%) | 25 (28.41) | 22 (26.83) | 0.865 |
| Local adverse events | | | |
| Pain, n (%) | 21 (23.86) | 17 (20.73) | 0.713 |
| Swelling, n (%) | 14 (15.91) | 11 (13.41) | 0.671 |
| Redness, n (%) | 11 (12.50) | 10 (12.20) | 1.000 |
| Itch, n (%) | 12 (13.64) | 7 (8.54) | 0.337 |
| Induration, n (%) | 11 (12.50) | 7 (8.54) | 0.461 |
| Systemic adverse events | | | |
| Muscle pain, n (%) | 21 (23.86) | 18 (21.95) | 0..856 |
| Pruritus, n (%) | 2 (2.27) | 1 (1.22) | 1.000 |
| Rash, n (%) | 1 (1.14) | 2 (2.44) | 0.610 |
| Fatigue, n (%) | 1 (1.14) | 0 (0) | 1.000 |
| Drowsiness, n (%) | 1 (1.14) | 0 (0) | 1.000 |
| Dizziness, n (%) | 15 (17.05) | 8 (9.76) | 0.185 |
| Headache, n (%) | 8 (9.09) | 7 (8.54) | 1.000 |
| Rhinorrhea, n (%) | 2 (2.27) | 1 (1.22) | 1.000 |
| Laryngeal pain, n (%) | 1 (1.14) | 0 (0) | 1.000 |
| Fever, n (%) | 1 (1.14) | 0 (0) | 1.000 |
| Chill, n (%) | 1 (1.14) | 0 (0) | 1.000 |
| Cough, n (%) | 0 (0) | 0 (0) | 1.000 |
| Inappetence, n (%) | 0 (0) | 0 (0) | 1.000 |
| Abdominal pain, n (%) | 0 (0) | 0 (0) | 1.000 |
| Abdominal distension, n (%) | 1 (1.14) | 0 (0) | 1.000 |
| Diarrhea, n (%) | 2 (2.27) | 0 (0) | 0.498 |
| Hepatalgia, n (%) | 0 (0) | 0 (0) | 1.000 |
| Nausea, n (%) | 0 (0) | 1 (1.22) | 0.482 |
| Chest distress, n (%) | 1 (1.14) | 0 (0) | 1.000 |
| Constipation | 0 (0) | 1 (1.22) | 0.482 |
