# Supplementary material for: Short-term safety and immunogenicity of inactivated and peptide-based SARS-CoV-2 vaccines in patients with endocrine-related cancer
Source: Front Immunol. 2022 Oct 24;13:1028246. doi: 10.3389/fimmu.2022.1028246 (PMC9637626; doi:10.3389/fimmu.2022.1028246)
Supplement: Supplementary file 2 [file Table_2.docx]

| Supplementary Table 2. Simple and multiple regression analysis to identify risk factors of low NAbs titers in endocrine-related cancer patients. | | | | |
| --- | --- | --- | --- | --- |
| Variables | Simple linear regression | | Multiple linear regression | |
|  | β value (95% CI) | P-value | β value (95% CI) | P-value |
| Age (years) | -0.021 (-0.031, 0.012) | 0.083 |  |  |
| Gender (female) | -0.207 (-0.311, 0.215) | 0.306 |  |  |
| **Days after full-course vaccination** | **-0.028 (-0.011, -0.009)** | **0.000** | **-0.032 (-0.022, -0.008)** | **0.000** |
| Comorbidity(ies) (no) | -0.031 (-0.033, 0.007) | 0.421 |  |  |
| **TNM (TNM 4)** | **0.211 (0.123, 0.999)** | **0.015** | **0.207 (1.183, 2.001)** | **0.015** |
| **ASA (ASA 3)** | **1.231 (1.033, 2.110)** | **0.025** | **1.178 (1.303, 2.321)** | **0.035** |
| Active treatment (no) | 0.208 (0.203, 2.010) | 0.189 |  |  |
| **Active chemotherapy (no)** | **-0.213 (-0.302, -0.113)** | **0.013** | **-0.131 (-0.141, -0.019)** | **0.012** |
| TNM: Tumor Node Metastasis; ASA: American Society of Anesthesiologists. | | | | |
