# Supplementary material for: Short-term safety and immunogenicity of inactivated and peptide-based SARS-CoV-2 vaccines in patients with endocrine-related cancer
Source: Front Immunol. 2022 Oct 24;13:1028246. doi: 10.3389/fimmu.2022.1028246 (PMC9637626; doi:10.3389/fimmu.2022.1028246)
Supplement: Supplementary file 3 [file Table_3.docx]

Supplementary Table 3. Clinical treatment information for all patients.

| Number | Cancer Type | Treatment protocol |
| --- | --- | --- |
| 1 | Thyroid carcinoma | Surgery + I131 therapy |
| 2 | Thyroid carcinoma | Surgery + I131 therapy |
| 3 | Thyroid carcinoma | None |
| 4 | Thyroid carcinoma | None |
| 5 | Thyroid carcinoma | Surgery |
| 6 | Thyroid carcinoma | Surgery + I131 therapy |
| 7 | Thyroid carcinoma | Surgery |
| 8 | Thyroid carcinoma | Surgery |
| 9（Negative） | Thyroid carcinoma | Unilateral subtotal thyroidectomy |
| 10 | Thyroid carcinoma | Surgery + I131 therapy |
| 11 | Thyroid carcinoma | Surgery |
| 12 | Thyroid carcinoma | Surgery |
| 13 | Thyroid carcinoma | Surgery |
| 14 | Thyroid carcinoma | None |
| 15 | Thyroid carcinoma | Surgery |
| 16 | Thyroid carcinoma | None |
| 17（Negative） | Thyroid carcinoma | Endoscopic radical thyroidectomy + Levothyroxine sodium |
| 18 | Thyroid carcinoma | Surgery + I131 therapy |
| 19 | Thyroid carcinoma | None |
| 20（Negative） | Thyroid carcinoma | Radical thyroidectomy + Levothyroxine sodium |
| 21 | Thyroid carcinoma | Surgery + I131 therapy |
| 22 | Thyroid carcinoma | Surgery + I131 therapy |
| 23 | Thyroid carcinoma | None |
| 24 | Thyroid carcinoma | Surgery |
| 25 | Thyroid carcinoma | Surgery + I131 therapy |
| 26（Negative） | Thyroid carcinoma | Radical thyroidectomy + Levothyroxine sodium |
| 27 | Thyroid carcinoma | Surgery |
| 28（Negative） | Thyroid carcinoma | Radical thyroidectomy + I131 therapy + Levothyroxine sodium |
| 29 | Thyroid carcinoma | Surgery |
| 30 | Thyroid carcinoma | Surgery + I131 therapy |
| 31 | Thyroid carcinoma | None |
| 32 | Thyroid carcinoma | Surgery |
| 33 | Thyroid carcinoma | None |
| 34 | Thyroid carcinoma | Surgery + I131 therapy |
| 35 | Thyroid carcinoma | Surgery |
| 36 | Thyroid carcinoma | Surgery + I131 therapy |
| 37 | Thyroid carcinoma | None |
| 38 | Thyroid carcinoma | Surgery |
| 39 | Thyroid carcinoma | Surgery + I131 therapy |
| 40 | Thyroid carcinoma | Surgery |
| 41 | Thyroid carcinoma | Surgery + I131 therapy |
| 42 | Thyroid carcinoma | Surgery |
| 43 | Thyroid carcinoma | Surgery + I131 therapy |
| 44 | Thyroid carcinoma | Surgery |
| 45 | Thyroid carcinoma | Surgery + I131 therapy |
| 46 | Thyroid carcinoma | Surgery + I131 therapy |
| 47 | Thyroid carcinoma | Surgery + I131 therapy |
| 48 | Thyroid carcinoma | None |
| 49 | Breast carcinoma | None |
| 50 | Breast carcinoma | Surgery + endocrine therapy |
| 51（Negative） | Breast carcinoma | Breast-conserving surgery + SLNB + AC chemotherapy + Trastuzumab + Tamoxifen |
| 52 | Breast carcinoma | Surgery + chemotherapy + endocrine therapy + radiotherapy |
| 53 | Breast carcinoma | Surgery + chemotherapy + endocrine therapy |
| 54（Negative） | Breast carcinoma | AC neoadjuvant chemotherapy + Modified radical mastectomy + SLNB + T chemotherapy + anastrozole |
| 55 | Breast carcinoma | Surgery + chemotherapy + endocrine therapy |
| 56 | Breast carcinoma | Surgery + endocrine therapy |
| 57 | Breast carcinoma | None |
| 58 | Breast carcinoma | None |
| 59 | Breast carcinoma | None |
| 60（Negative） | Breast carcinoma | Radical mastectomy + anastrozole |
| 61 | Breast carcinoma | Surgery + chemotherapy + endocrine therapy |
| 62（Negative） | Breast carcinoma | Breast-conserving surgery + T chemotherapy |
| 63 | Breast carcinoma | Surgery + endocrine therapy |
| 64 | Breast carcinoma | Surgery + chemotherapy + endocrine therapy |
| 65（Negative） | Breast carcinoma | Radical mastectomy + SLNB + TC chemotherapy + Letrozole |
| 66 | Breast carcinoma | Surgery + endocrine therapy |
| 67 | Breast carcinoma | Surgery + endocrine therapy |
| 68 | Breast carcinoma | Surgery + chemotherapy + endocrine therapy + radiotherapy |
| 69（Negative） | Breast carcinoma | Radical mastectomy + SLNB + ACT chemotherapy + Leuprorelin + Letrozole + Radiotherapy |
| 70（Negative） | Breast carcinoma | ECT neoadjuvant chemotherapy + SLNB + Modified radical mastectomy + Capecitabine |
| 71 | Breast carcinoma | Surgery + endocrine therapy |
| 72 | Breast carcinoma | Surgery + endocrine therapy |
| 73 | Breast carcinoma | Surgery + targeted therapy + chemotherapy + endocrine therapy |
| 74 | Breast carcinoma | Surgery + chemotherapy |
| 75 | Breast carcinoma | Surgery + chemotherapy |
| 76 | Breast carcinoma | Surgery + chemotherapy |
| 77（Negative） | Breast carcinoma | TAC neoadjuvant chemotherapy |
| 78 | Breast carcinoma | Surgery + chemotherapy + endocrine therapy |
| 79（Negative） | Breast carcinoma | Breast-conserving surgery + TC chemotherapy |
| 80 | Breast carcinoma | Surgery + endocrine therapy |
| 81 | Breast carcinoma | Surgery + chemotherapy + endocrine therapy + radiotherapy |
| 82（Negative） | Breast carcinoma | ACT neoadjuvant chemotherapy + Modified radical mastectomy + Trastuzumab + Tamoxifen |
| 83（Negative） | Breast carcinoma | Pirarubicin neoadjuvant chemotherapy + Modified radical mastectomy + SLNB |
| 84 | Breast carcinoma | Surgery + chemotherapy + endocrine therapy |
| 85 | Breast carcinoma | Surgery + chemotherapy + endocrine therapy |
| 86 | Breast carcinoma | Surgery + endocrine therapy |
| 87 | Breast carcinoma | Surgery + targeted therapy + chemotherapy + endocrine therapy |
| 88 | Breast carcinoma | Surgery + endocrine therapy |
| SLNB: sentinel lymph node biopsy; AC: A Epirubicin hydrochloride + C Cyclophosphamide; T: Paclitaxel; TC: T Paclitaxel + C Cyclophosphamide; ECT: E Doxorubicin + C Cyclophosphamide + T Paclitaxel; ACT: A Epirubicin hydrochloride + C Cyclophosphamide + T: Paclitaxel; TAC: Docetaxel + A Epirubicin hydrochloride + C Cyclophosphamide; | | |
