# Supplementary figures and images for: Short-term safety and immunogenicity of inactivated and peptide-based SARS-CoV-2 vaccines in patients with endocrine-related cancer
Source: Front Immunol. 2022 Oct 24;13:1028246. doi: 10.3389/fimmu.2022.1028246 (PMC9637626; doi:10.3389/fimmu.2022.1028246)

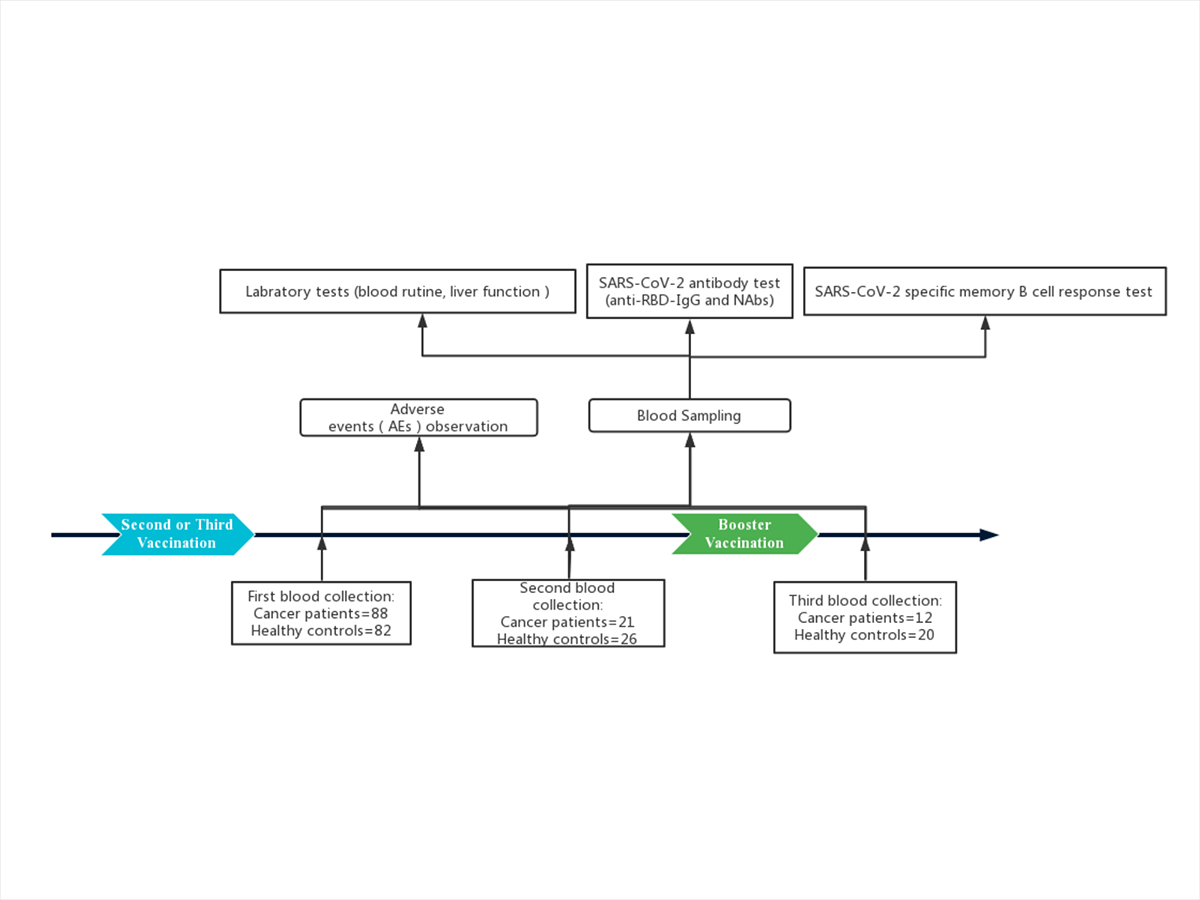

Supplement: Supplementary Figure 1 — Study flow chart. [file Image_1.tif]

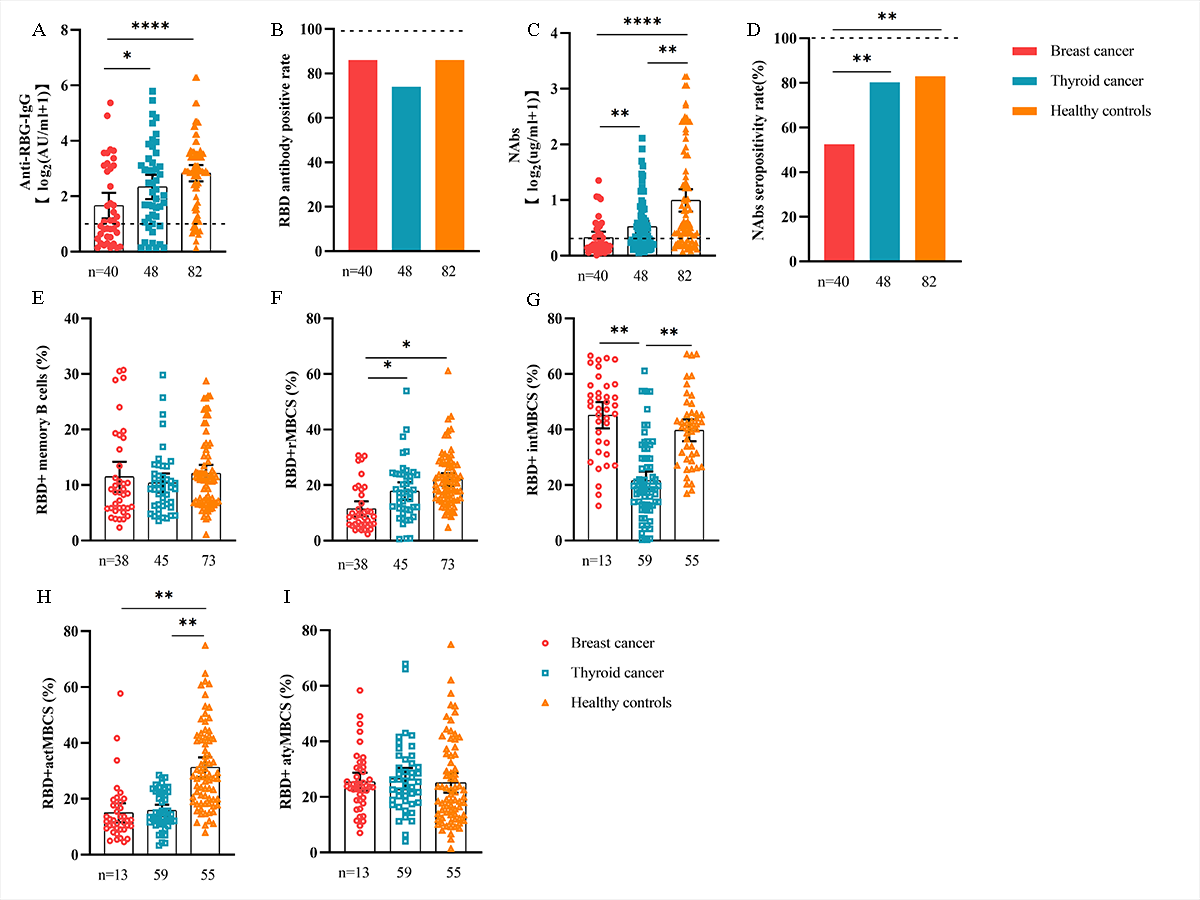

Supplement: Supplementary Figure 2 — Responses of antibodies and RBD+ B cells to the SARS-COV-2 vaccines. The responses of antibodies (A–D) and RBD+ MBCs (E–I) in breast or thyroid cancer and healthy controls. RBD, receptor-binding domain, NAbs, neutralizing antibodies, MBCs, memory B cells. [file Image_2.tif]

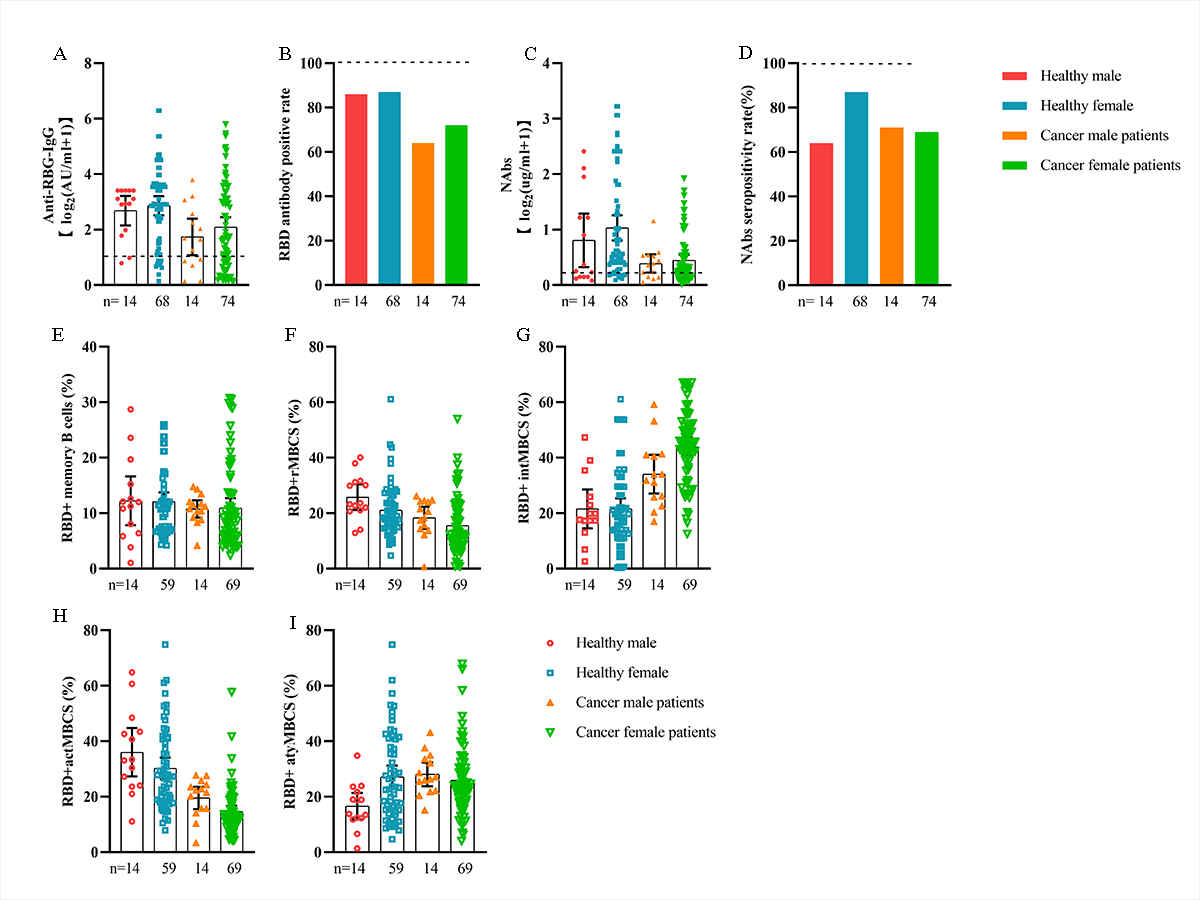

Supplement: Supplementary Figure 3 — Responses of antibodies and RBD+ B cells to the SARS-COV-2 vaccines. Responses of antibodies (A–D) and RBD+ MBCs (E–I) in patients with ER cancer and healthy controls stratified by sex. ER, endocrine-related, RBD, receptor-binding domain, NAbs, neutralizing antibodies, MBCs, memory B cells. [file Image_3.tif]

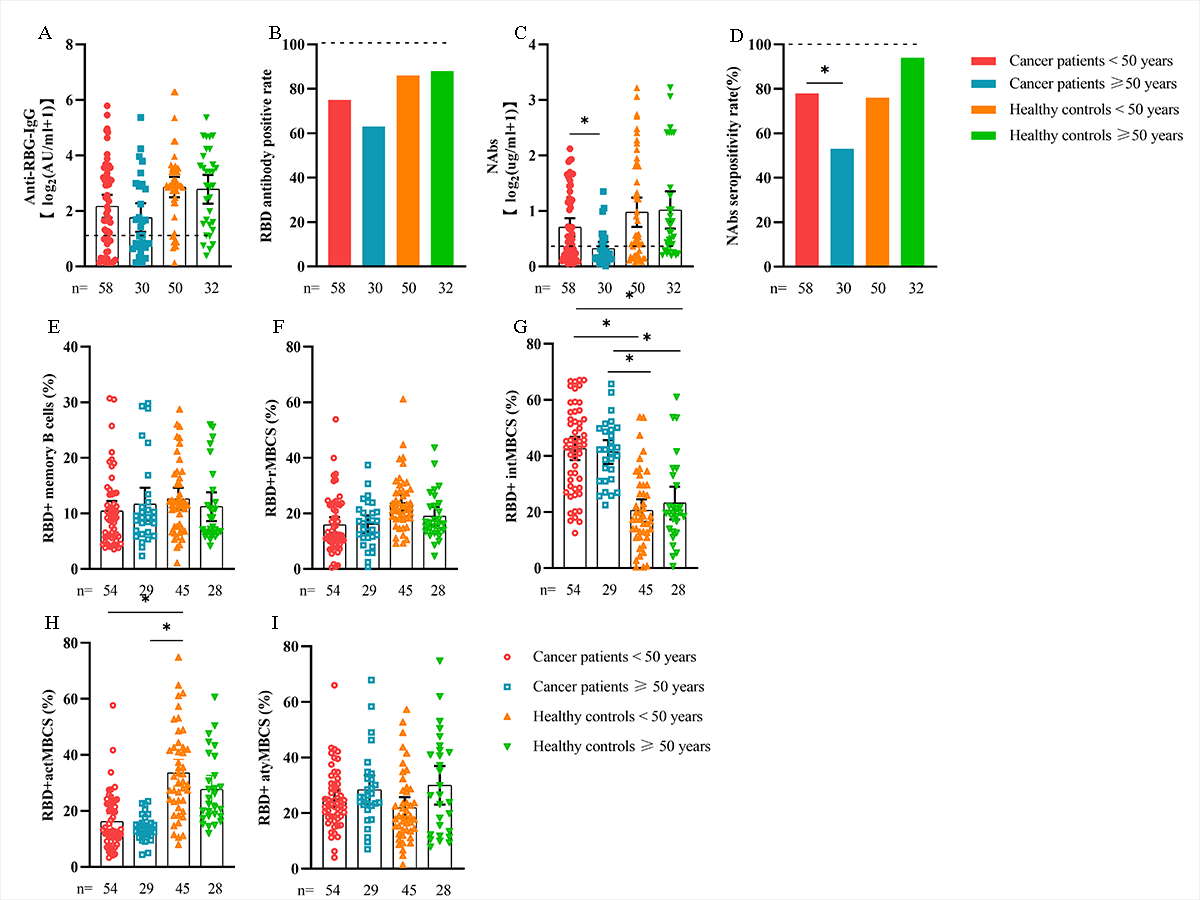

Supplement: Supplementary Figure 4 — Responses of antibodies and RBD+ B cells to the SARS-COV-2 vaccines. The responses of antibodies (A–D) and RBD+ MBCs (E–I) in patients with ER cancer stratified by age. ER, endocrine-related, RBD, receptor-binding domain, NAbs, neutralizing antibodies, MBCs, memory B cells. [file Image_4.tif]

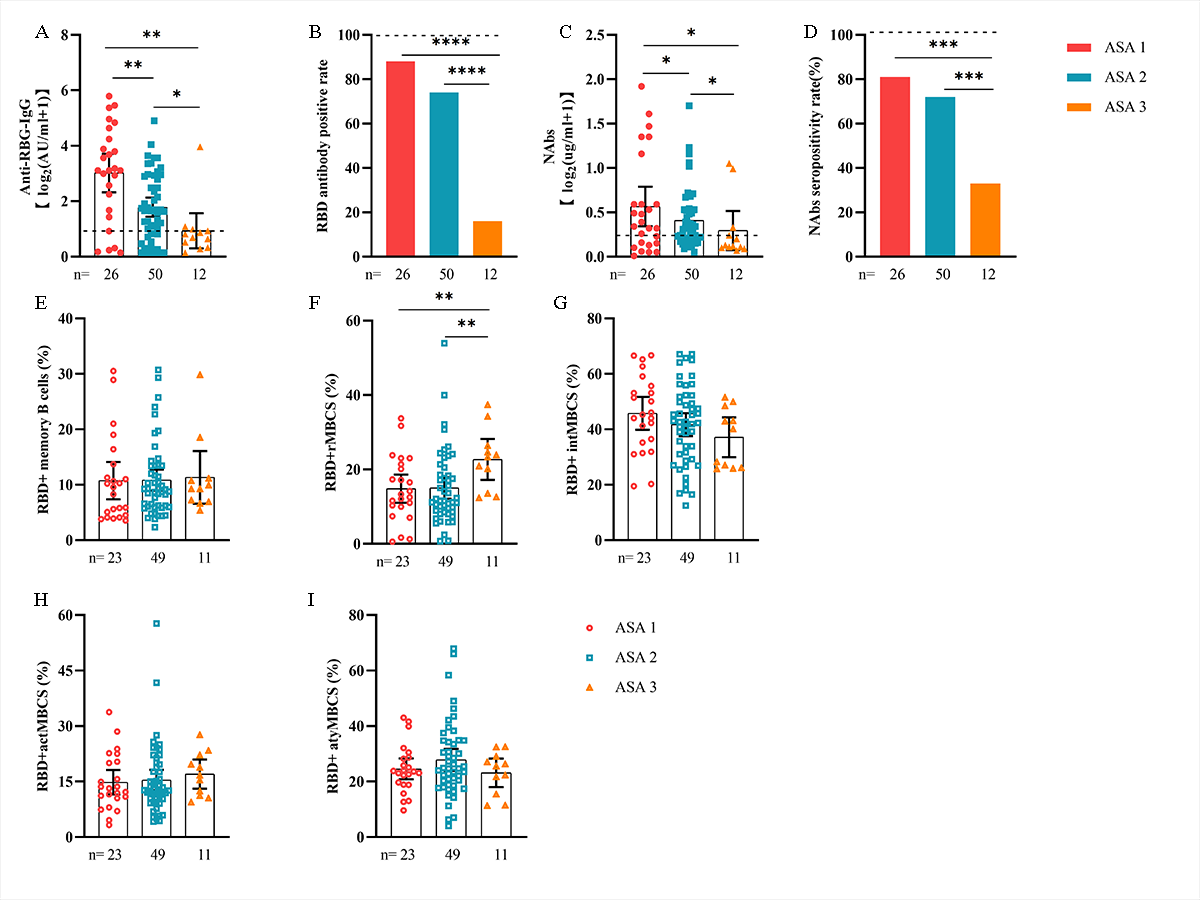

Supplement: Supplementary Figure 5 — Responses of antibodies and RBD+ B cells to the SARS-COV-2 vaccines. Responses of antibodies (A–D) and RBD+ MBCs (E–I) in patients with ER cancer stratified by ASA scores. ER, endocrine-related, RBD, receptor-binding domain, NAbs, neutralizing antibodies, MBCs, memory B cells. [file Image_5.tif]

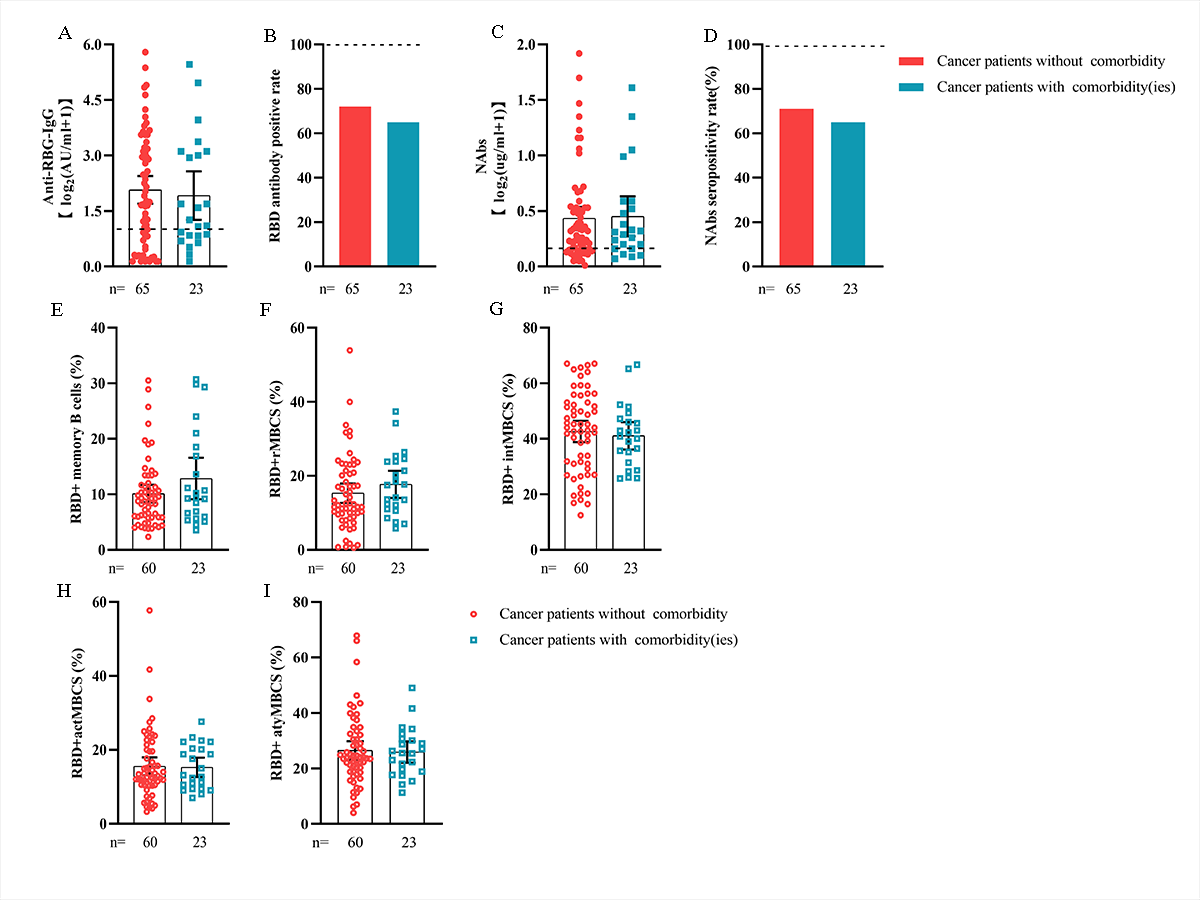

Supplement: Supplementary Figure 6 — Responses of antibodies and RBD+ B cells to the SARS-COV-2 vaccines. Responses of antibodies (A–D) and RBD+ MBCs (E–I) in patients with ER cancer with/without comorbidity(ies). ER, endocrine-related, RBD, receptor-binding domain, NAbs, neutralizing antibodies, MBCs, memory B cells. [file Image_6.tif]

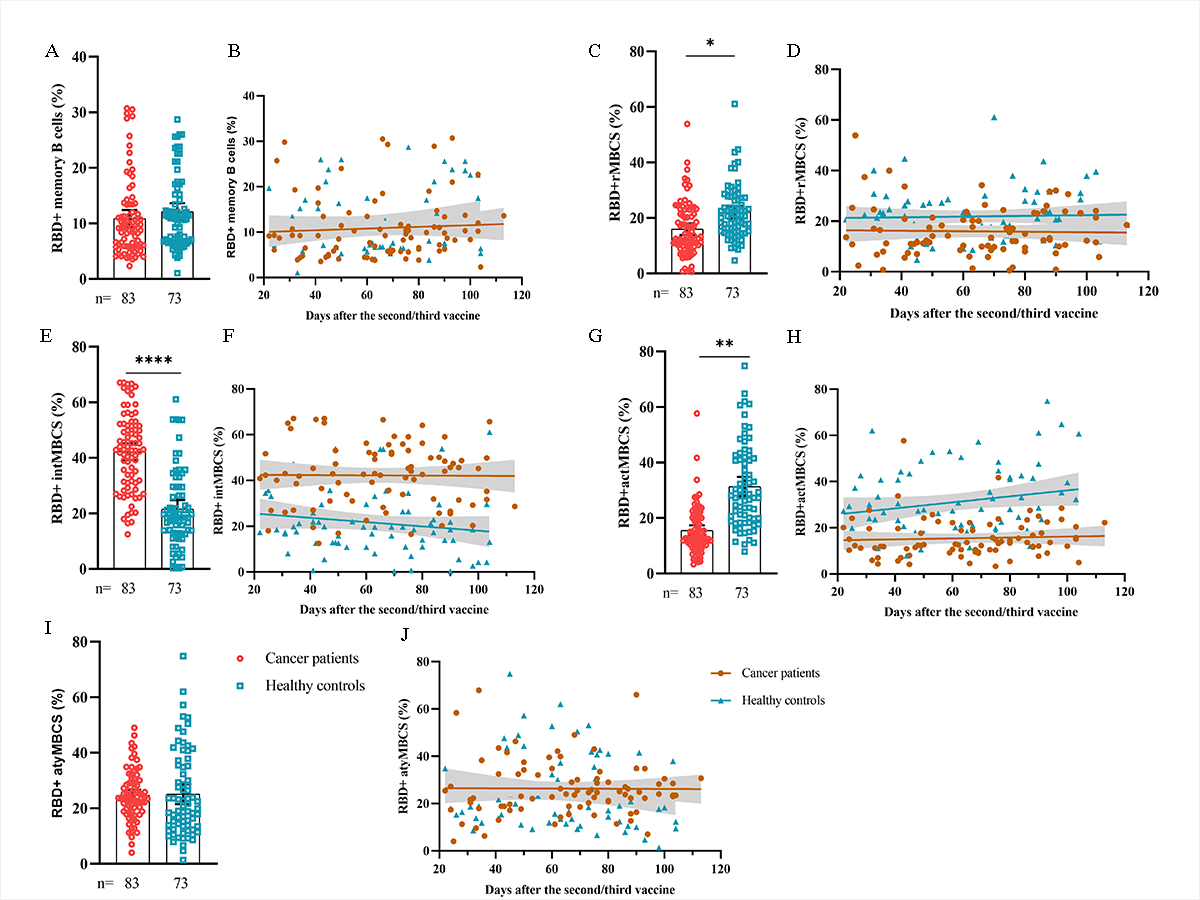

Supplement: Supplementary Figure 7 — Responses of RBD+ B cells. The frequencies of RBD+ MBCs in ER cancer patients and healthy controls (A, C, E, G, I). The change of frequencies of RBD+ MBCs (B, D, F, H, J) over time in ER cancer patients and healthy controls. ER, endocrine-related, RBD, receptor-binding domain, MBCs, memory B cells. [file Image_7.tif]

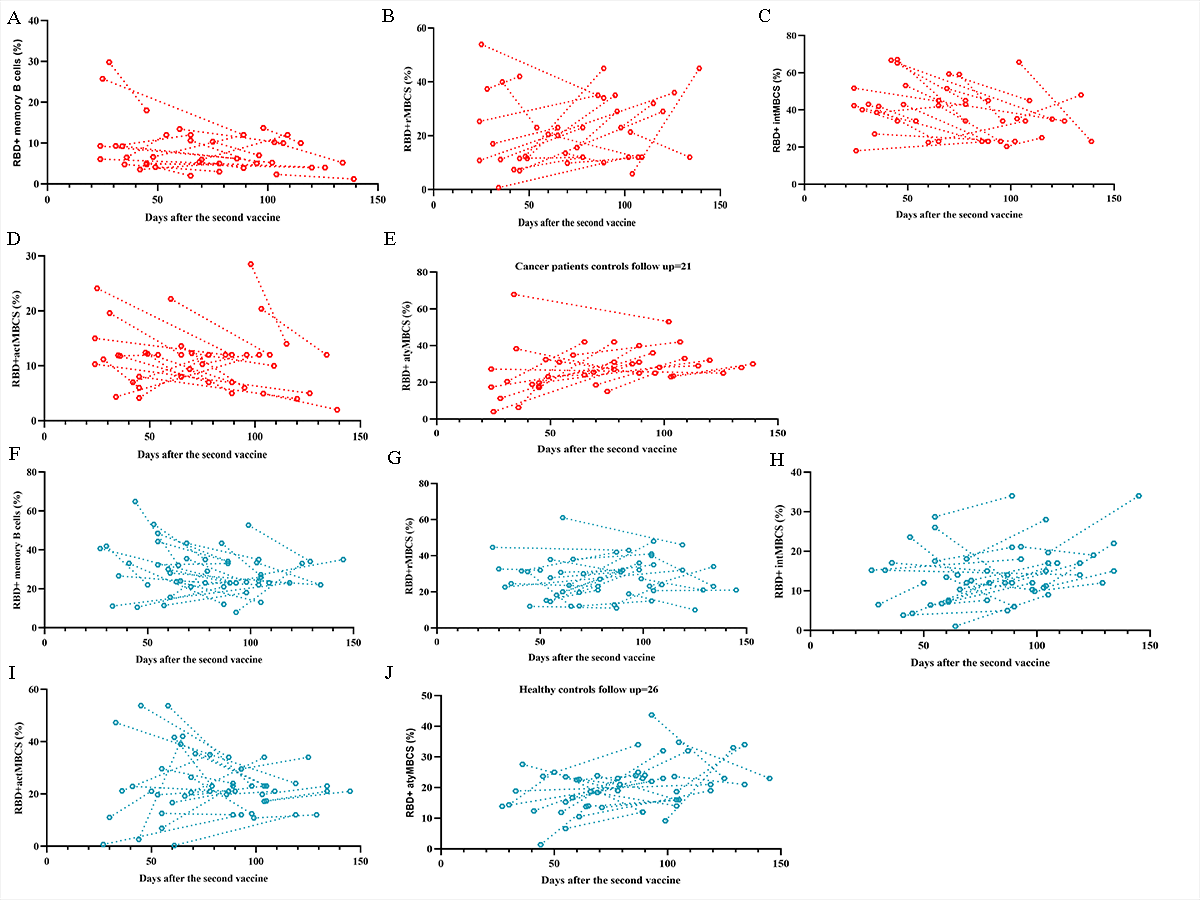

Supplement: Supplementary Figure 8 — Responses of RBD+ B cells over time. Dynamic changes of RBD+ MBCs and their subpopulations over time in ER patients and healthy controls. The red dots represent patients with ER cancer, and the blue dots represent healthy controls. RBD, receptor-binding domain, MBC, memory B cells, ER, endocrine related. [file Image_8.tif]

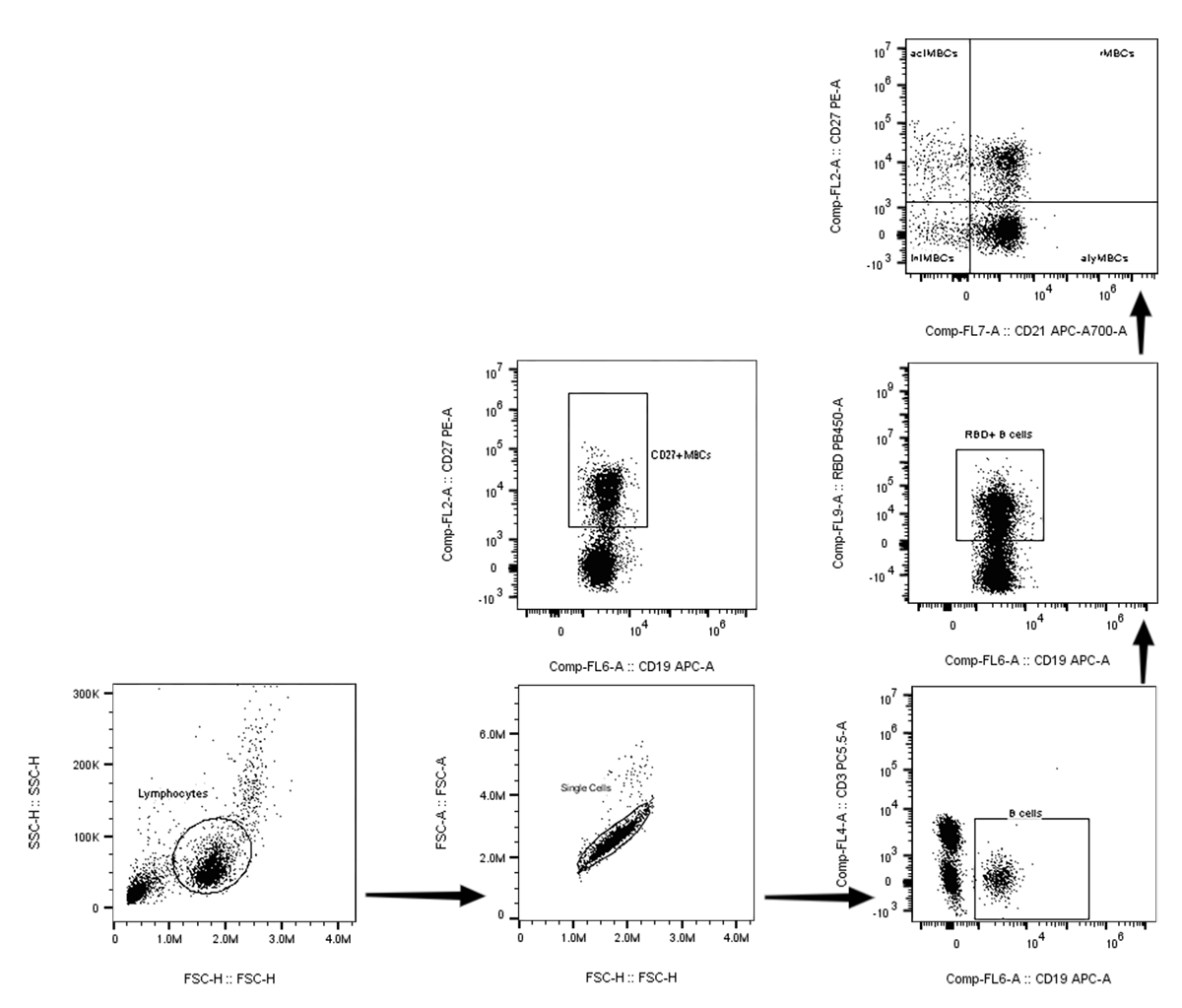

Supplement: Supplementary Figure 9 — Gating strategy for the target cell population. RBD, receptor-binding domain. [file Image_9.tif]
